# Experimental Stroke Differentially Affects Discrete Subpopulations of Splenic Macrophages

**DOI:** 10.3389/fimmu.2018.01108

**Published:** 2018-05-22

**Authors:** Laura McCulloch, Alessio Alfieri, Barry W. McColl

**Affiliations:** ^1^UK Dementia Research Institute, Edinburgh Medcial School, University of Edinburgh, Edinburgh, United Kingdom; ^2^Centre for Discovery Brain Sciences, University of Edinburgh, Edinburgh, United Kingdom

**Keywords:** stroke, immunosuppression, spleen, macrophages, infection

## Abstract

Changes to the immune system after stroke are complex and can result in both pro-inflammatory and immunosuppressive consequences. Following ischemic stroke, brain resident microglia are activated and circulating monocytes are recruited to the injury site. In contrast, there is a systemic deactivation of monocytes/macrophages that may contribute to immunosuppression and the high incidence of bacterial infection experienced by stroke patients. The manipulation of macrophage subsets may be a useful therapeutic strategy to reduce infection and improve outcome in patients after stroke. Recent research has enhanced our understanding of the heterogeneity of macrophages even within the same tissue. The spleen is the largest natural reservoir of immune cells, many of which are mobilized to the site of injury after ischemic stroke and is notable for the diversity of its functionally distinct macrophage subpopulations associated with specific micro-anatomical locations. Here, we describe the effects of experimental stroke in mice on these distinct splenic macrophage subpopulations. Red pulp (RP) and marginal zone macrophages (MZM) specifically showed increases in density and alterations in micro-anatomical location. These changes were not due to increased recruitment from the bone marrow but may be associated with increases in local proliferation. Genes associated with phagocytosis and proteolytic processing were upregulated in the spleen after stroke with increased expression of the lysosome-associated protein lysosomal-associated membrane proteins specifically increased in RP and MZM subsets. In contrast, MHC class II expression was reduced specifically in these populations. Furthermore, genes associated with macrophage ability to communicate with other immune cells, such as co-stimulatory molecules and inflammatory cytokine production, were also downregulated in the spleen after stroke. These findings suggest that selective splenic macrophage functions could be impaired after stroke and the contribution of macrophages to stroke-associated pathology and infectious complications should be considered at a subset-specific level. Therefore, optimal therapeutic manipulation of macrophages to improve stroke outcome is likely to require selective targeting of functionally and spatially distinct subpopulations.

## Introduction

Stroke induces rapid and complex effects on the systemic immune system. Immune cells including monocytes/macrophages infiltrate the brain after stroke and original studies suggested that they exacerbate inflammation and injury (1–3). However, prevention of monocyte/macrophages from entering the brain in the acute phase after ischemic stroke has been shown to worsen outcome, and we now know that certain subpopulations can be beneficial for injury repair and recovery (4–7). Conversely, there is a concurrent suppression of systemic immune responses including loss of B and T lymphocytes (8, 9), atrophy of immune organs (10) and functional deactivation of monocytes (11, 12). This contributes to a susceptibility to infection, which is the most common complication of stroke. Stroke-associated infection (SAI) affects up to one third of patients and is independently associated with increased mortality and morbidity (13, 14). Reducing infection in stroke patients may provide a useful strategy to improve clinical outcome; however, successful therapeutics must restore peripheral immune defenses without exacerbating harmful brain inflammation and injury. The spleen has been identified as an important reservoir of monocytes which are mobilized in response to inflammation (15). Furthermore, the spleen undergoes extensive atrophy due to apoptosis of resident immune cell populations after experimental and clinical stroke which is potentially compounded by neural stimulation of the smooth muscle surrounding the capsule inducing splenic contraction (8, 10, 14, 16). For this reason, splenic macrophages may have multiple contributions to the neuroimmune mechanisms influencing stroke and have been identified as a potential therapeutic target for both preventing excessive harmful inflammation in the brain and reducing immunosuppression after stroke (5, 17).

The spleen is comprised of two functionally distinct compartments. The red pulp (RP) functions as a blood filter to remove dead erythrocytes, acts as a storage site for erythrocytes, platelets, and iron, and in rodents is a site of hematopoiesis. The white pulp (WP) is a highly organized immune compartment crucial in the defense against systemic infection and is surrounded by a marginal zone (MZ), which acts as an interface between the circulation and cells of the immune system (18). Specific populations of macrophages exist in each of these functionally distinct compartments. Splenic macrophages are a heterogeneous population with respect to origin, micro-anatomical location, and surface receptor expression but contribute in complementary ways to maintain homeostasis, reduce blood-borne infection, and induce effective immune responses.

Red pulp macrophages (RPM) in the spleen can be divided into F4/80^hi^ CD11b^−/lo^ and F4/80^+^ CD11b^+^ populations with distinct developmental lineages and functions (19). F4/80^hi^ CD11b^−/lo^ comprise the majority of RPM and are a CSF-1-dependent, self-renewing, tissue resident macrophage population derived from the fetal liver (19, 20). In contrast, F4/80^+^ CD11b^+^ RPM are monocyte-derived macrophages and have a smaller contribution to the RPM pool. These cells are replenished from bone marrow hematopoietic precursors at low levels in steady state but can aid replenishment of tissue-resident RPM in conditions that induce macrophage depletion and as such express the chemokine receptor CCR2 (19, 21, 22). Both populations are responsible for the clearance of senescent red blood cells and degrade hemoglobin to transport the free iron back into the circulation (23). These functions are crucial for systemic iron metabolism and the removal of potentially harmful self-antigen from dying cells in the circulation but are additionally important for antibacterial defenses through the sequestration of free iron to limit bacterial growth. RPM can also differentially influence the development of adaptive immune responses. F4/80^+^ CD11b^+^ RPM are reported to have potent antigen-presenting capabilities, produce pro-inflammatory cytokines, and can induce T cell proliferation *in vitro*, whereas F4/80^hi^ CD11b^−/lo^ RPM produced anti-inflammatory cytokines and induced the differentiation of regulatory T cells thought to be important for regulation of autoimmune responses to self-antigen filtered in the RP (19, 21).

Arterial blood entering the spleen interacts with two populations of non-motile macrophages in the MZ allowing surveillance of pathogens in the circulation. Localization of marginal zone macrophages (MZM) and marginal metallophillic macrophages (MMM) at the interface between the circulation and organized lymphoid tissue in the WP allow them to act as a bridge between the innate and adaptive immune responses. The origin of macrophages in the MZ is currently uncertain but mice deficient in the nuclear receptor LXRα have a specific deficiency in MMM and MZM which is restored upon transfer of WT monocytes suggesting they can be replenished from bone marrow-derived progenitors (24). MZM line the marginal sinus on the external side and are defined by expression of the macrophage scavenger receptor MAcrophage Receptor with a COllagenous structure, MARCO (25). MMM are localized at the inner side of the marginal sinus forming a ring around the periarteriolar lymphocyte sheath (PALS) and follicular areas of the WP and can be distinguished using the monoclonal antibody MOMA-1 (CD169) (26). MZ macrophage populations express a variety of pattern recognition receptors (PRR) allowing efficient recognition and clearance of pathogens crucial in the defense against systemic infection. The clearance of viral, parasitic, and bacterial pathogens by MZ macrophages has been shown to be essential for early containment of infection (27–29). MMM uniquely express nonspecific esterase, which is important in the neutralization of LPS (30). Additionally, both MZ macrophage populations have roles in the clearance of apoptotic cells and suppressing immune responses to self-antigen present in cellular debris (31). MARCO^+^ MZM in the spleen can regulate B cell transport of antigen to the follicle (32). MOMA-1^+^ MMM can transport bacteria to splenic WP to allow subsequent waves of innate immune responses and an analogous MOMA-1^+^ macrophages in the subcapsular sinus of the lymph node have been shown to directly transport antigen to the follicular dendritic cells (FDC) (33, 34), processes that are crucial in the germinal center (GC) reaction to allow the development of high affinity antibody responses.

Within the WP, tingible body macrophages (TBM) are found in the B cell follicles and are responsible for the clearance of apoptotic lymphocytes that arise as the result of the GC reaction (35). TBM clearance of apoptotic cells is dependent on MFGE-8 produced by FDC and defects in these pathways are also linked to increased autoimmune responses (36). Dendritic cells (DC) are also present in the WP and MZ and share some similar functions to macrophages but are specialized for antigen collection, processing, and transport to secondary lymphoid organs for presentation to T cells (37, 38). DC in the MZ (MZ DC) have additional abilities and can prime B cells to initiate antibody responses and regulate peripheral tolerance to self-antigen (39, 40).

In the present study, we determined the effects of experimental stroke on these individual populations and analyzed their location and density within the splenic micro-environment. We have shown that splenic macrophage subpopulations were differentially affected by experimental stroke and that increased local densities of both subsets of RPM, and MZM did not result from increased recruitment of precursors from the bone marrow but may instead be associated with increases in local proliferation. Furthermore, the same subsets show evidence supporting functional changes including increased phagocytosis and reduced expression of MHC Class II after experimental stroke. Genome-wide analysis of splenocytes after stroke indicated that genes associated with macrophage ability to detect infection and recruit and activate other immune cells were also reduced after stroke including those encoding expression of PRR, co-stimulatory molecules, and pro-inflammatory cytokines. These data suggest further investigations of the functional consequences of stroke at a subpopulation-specific level are warranted and will provide the optimal basis to identify therapeutic targets to augment macrophage function and reduce SAI without exacerbating their potentially brain-damaging actions or compromising pro-repair mechanisms within the injured brain.

## Materials and Methods

### Animals

Male 8–10-week-old C57BL/6J mice were purchased (Charles River Laboratories, UK) and used in all experiments unless otherwise stated. CCR2^RFP/+^ transgenic mice were used where stated and have been previously described (41). Breeding pairs of CCR2^RFP/+^ reporter mice on a C57BL/6J background, were purchased from Jackson Laboratories (ME, USA) and a colony was established in house. Mice were maintained under SPF conditions and a standard 12-h light/dark cycle with unrestricted access to food and water. Mice were housed in individually ventilated cages in groups of up to five mice and those bought from external suppliers were acclimatized for a minimum of 1 week prior to procedures. All animal experiments were carried out under the authority of a UK Home Office Project License in accordance with the “Animals (Scientific procedures) Act 1986” and Directive 2010/63/EU and were approved by both The Roslin Institute’s and the University of Edinburgh’s Animal Welfare and Ethics Review Board. Experimental design, analysis, and reporting followed the ARRIVE guidelines (https://www.nc3rs.org.uk/arrive-guidelines).

### Experimental Stroke Model and Tissue Harvesting

Middle cerebral artery occlusion (MCAO) was performed under isoflourane anesthesia (with O_2_ and N_2_O) by insertion of a 6-0 nylon monofilament with a 2 mm coated tip (210 µm diameter; Doccol, USA) through the external carotid artery and advanced through the internal carotid artery to occlude the MCA. The filament was withdrawn after 40 min to allow reperfusion, the neck wound sutured, and the animals recovered. Topical local anesthetic (lidocaine/prilocaine) was applied to the wound. For sham surgery, the filament was advanced to the MCA and immediately retracted. Sham-operated animals remained anesthetized for 40 min and recovered as above. Core body temperature was maintained at 37 ± 0.5°C throughout the procedure with a feedback controlled heating blanket (Harvard Aparatus). Animals were recovered for 1–7 days then anesthetized and anti-coagulated (3.8% w/v tri-sodium citrate) cardiac blood samples were taken followed by transcardiac perfusion with saline. Brains were harvested and frozen in isopentane at −35 to −45°C over dry ice. Spleens used for flow cytometric analysis were harvested into ice cold PBS. For immunohistochemical analysis, spleens from C57Bl/6 mice were snap frozen on dry ice and all tissues were stored at −80°C until sections cut. Spleens from *Ccr2*^RFP/+^ reporter mice were perfused with saline and immersion fixed overnight in 4% paraformaldehyde. Samples were rinsed in dH_2_O and immersed in 20% sucrose at 4°C for 24 h. Excess sucrose was removed from sample by blotting on absorbent tissue and samples were frozen in isopentane at −35 to −45°C over dry ice and stored at −80°C until sections cut.

### Immunohistochemistry and Analysis of Spleens

Serial frozen sections of spleen (6 µm in thickness) were cut on a cryostat, fixed in ice-cold acetone for 10 min, washed in 0.05% BSA in PBS, and blocked using species-specific normal serum (Jackson Immunoresearch Laboratories Inc., PA, USA) according to secondary antibody used. Primary antibodies were incubated for 1 h at room temperature. Splenic macrophage populations were visualized by staining for F4/80 (1 µg/ml; clone BM8, Biolegend, CA, USA), CD11b (5 µg/ml; clone M1/70, eBioscience, ThermoFisher Scientific, Paisley, UK), MOMA-1 to detect CD169 (2.5 µg/ml; Siglec 1; clone 3D6.112, Biolegend), MARCO (10 µg/ml; clone ED31, AbD Serotec, Kidlington, UK), MOMA-2 (1 µg/ml; clone MOMA2; Abcam, Cambridge, UK) or CD68 (5 µg/ml; clone FA-11; Biolegend). Monocytes/Granulocytes were visualized by staining for Ly6C (1 µg/ml; clone HK1.4; Biolegend). Neutrophils were visualized using Ly6G (1 µg/ml; clone 1A8; Biolegend) and SJC4 (1/10,000; polyclonal; Anthony lab, Oxford). DC populations were visualized by staining for CD11c (1 µg/ml; clone N4/18, Biolegend) and DEC205 (5 µg/ml; clone NLDC-145, Biolegend). Proliferating cells were visualized by staining with KI-67 (1 µg/ml; clone 16A8, Biolegend). Macrophage-associated proteins were visualized by staining for MHC II to detect IA/IE (0.5 µg/ml; clone M5/114/15.2, Biolegend) and lysosomal-associated membrane proteins (LAMP2) to detect CD107b (0.5 µg/ml; clone M3/84, Biolegend). Following the addition of primary antibody, sections were washed in PBS-BSA. For unconjugated primary antibodies, 2.5 µg/ml of species-specific secondary antibody coupled to Alexa Fluor^®^ 488, Alexa Fluor^®^ 594 dyes or Alexa Fluor^®^ 647 fluorochromes (Life Technologies Ltd, Paisley, UK) were incubated for 1 h at room temperature. Sections were washed in PBS-BSA and mounted in fluorescent mounting medium (Dako, Cambridgeshire, UK) and images captured using a Zeiss LSM5 confocal microscope (Zeiss, Welwyn Garden City, UK).

Digital images were analyzed using ImageJ software. All spleens from each experimental group were analyzed. From each spleen, two sections, 100 µm apart, were studied and on each section data from four individual areas of WP were collected. Fluorescent intensity thresholds were applied, and the number of pixels of each color (black, red, green, yellow) was automatically counted as previously described (42) and used to determine the area of immunolabelling for each antibody. To analyze MZ specific immunolabelling, a border was firstly drawn to separate the MZ from the follicle using MZ macrophage immunolabelling as a guide. The area of WP and MZ zone specific immunolabelling was determined separately.

### Flow Cytometry

Mouse spleens were mechanically dissociated and red blood cells were lysed. Resulting single cell suspensions were used for flow cytometric analysis of macrophage populations and CCR2-RFP expression. Briefly, cell surface staining to allow the detection of macrophage subpopulations was carried out using antibodies against F4/80-PercP-Cy5 (clone BM8; Biolegend, 1 µg/ml), CD11b-APC-Cy7 (clone M1/70; Biolegend, 1 µg/ml), MOMA-1-PE (CD169; clone3D6.112; Biolegend, 2 µg/ml), and MARCO (clone ED31; Bio-rad, 5 µg/ml). Cells were incubated in anti-mouse CD16/32 (clone 93; Affymetrix eBioscience, 0.4 µg/ml) for 10 min to block Fc receptors then fluorescently conjugated primary antibody cocktails for 30 min at room temperature. Cells were washed in PBS and flow cytometry was performed on a Becton Dickinson LSR Fortessa and analyzed by Summit software (Dako). Live cells were gated based on FSC and SSC and 20,000 cells in this gate collected for each sample.

### Microarray

Microarray analysis combined previously published analysis of sham and 5 days post-MCAO datasets (NCBI Geodataset database accession number GSE70841) with further unpublished datasets from mice recovered 1 and 2 days after MCAO to provide a new analysis. RNA was isolated from 1/4 spleens from sham-operated C57Bl/6J mice or those recovered from MCAO for 1, 2, or 5 days using the Qiagen RNeasy mini kit (Qiagen, Manchester, UK). RNA integrity and quality were verified using Agilent BioAnalyzer 2100 (Agilent Technologies, Santa Clara, CA, USA). RNA was reverse-transcribed to cDNA, amplified, and labelled using the Affymetrix terminal labeling kit (Affymetrix, Santa Clara, CA, USA), according to the manufacturer’s instructions. Samples were hybridized to MoGene 1.0 ST chips (Affymetrix) using GeneTitan Hybridization, Wash and Stain kit for WT array plates (Affymetrix), stained, and scanned by Edinburgh Genomics (Edinburgh, UK). Raw data (.cel) files were normalized directly during import for analysis in Partek Genomics Suite (Partek Inc, MO, USA). Probesets were annotated using MoGene-1_0-st-v1 Probeset Annotations, Release 35. To determine which transcripts were changed due to experimental stroke, normalized datasets were compared in Transcriptome Analysis Console (Affymetrix) by ANOVA. Expression of specific transcripts of interest was determined using normalized raw data in excel. Microarray data are deposited in the NCBI GeoDatasets database with the accession number GSE108875.

### Cytometric Bead Array

Cytometric bead array was used to measure splenic cytokine concentrations. 1/4 spleens were homogenized to a 20% weight/volume concentration in buffer containing 50 mM Tris-HCl pH6.7; 150 mM NaCl; 5 mM CaCl_2_; 1% TritonX; and 1× protease inhibitor cocktail set 1 (Merck, Calbiochem, Hertfordshire, UK). BD™ CBA flex Set containing mouse cytometric bead arrays for IL-1α; IL-4; IL-6; IL-10; G-CSF; CCL-2; CXCL-1; and TNFα was used according to manufacturer’s instructions (BD Biosciences, Oxford, UK). Briefly, spleen homogenates were diluted 1:1 in assay diluent, standards were prepared according to manufacturer’s instructions and both were added to plate with a cocktail of cytokine-specific beads and incubated for 1 h on a shaker. PE-conjugated cytokine-specific secondary antibody cocktail was prepared according to manufacturer’s instructions, added to plate, and incubated for a further 1 h. Beads were washed and then resuspended in PBS. Beads were acquired on a BD FACS Array cytometer and data were analyzed using FCAP Array™ Software.

### Splenic MZ Antigen Capture

To assess the capture and transport of antigen by MZ cells *in vivo*, naïve mice or mice recovered 48 h after sham or MCAO, were administered 20 µg of 3,000 kDa Dextran conjugated to FITC (Sigma, Dorset, UK) by intravenous injection. Spleens were removed either 1 or 24 h after injection, snap frozen on dry ice and 5 µm sections cut on a cryostat. The presence of MZ cell-associated or FDC-associated fluorescent antigen was identified by immunohistochemistry and analyzed in ImageJ as described above.

### Quantitative Real-Time Polymerase Chain Reaction (qRT-PCR)

500 ng of total RNA prepared for microarray (as above) was reverse–transcribed using superscript III Reverse transcriptase (Life technologies) according to manufacturer’s instructions. RT-qPCR was carried out on a Stratagene Mx3005P instrument (Agilent Technologies) using either RT^2^ SYBR^®^ Green qPCR mastermix (Qiagen) and RT^2^ qPCR primer assays for *Gapdh, Tlr1, Cd40, Tnf, Lamp2*, and *Ctsl* or Platinum SYBR^®^ Green qPCR SuperMix-UDG (Invitrogen, USA) and KiCqStart™ SYBR^®^ Green primers (Sigma) for *Cd163* and *Lcn2* as detailed in Tables 1 and 2, respectively, according to manufacturer’s instructions. qPCR cycles were performed as follows: hot-start denaturation cycle 95°C for 10 min, 40 cycles of amplification of 95°C for 15 s and 60°C for 1 min ΔCt (Ct of housekeeping gene, *Gapdh*, minus Ct of gene of interest) was calculated for each sample. Data are expressed as the normalized expression ratio (2^(−ΔΔCt)^) in comparison to sham samples.

**Table 1:** RT^2^ qPCR primer assays (no sequence available).

Protein	Gene symbol	Gene ID	Reference position	Catalog number
TNFα	Tnf	21926	194 (NM_013693)	PPM0113G
CD40	Cd40	21939	885 (NM_011611)	PPM03426C
Cathepsin L	Ctsl	13039	898 (NM_009984)	PPM03691C
GAPDH	Gapdh	14433	478 (NM_008084)	PPM02946E
Lamp2	Lamp2	16784	1152 (NM_001017959)	PPM24540E
TLR 1	Tlr1	21897	2334 (NM_030682)	PPM04211B

**Table 2:** KiCqStart SYBR Green primers.

Protein	Gene symbol	Gene ID	Sequence 5′- 3′ F	Sequence 5′- 3′ R
Lipocalin2	Lcn2	16819	ATATGCACAG GTATCCTCAG	GAAACGTTCCT TCAGTTCAG
CD163	Cd163	93671	AGTCTGCTCAC GATACATAG	TCCTTCTGGAAT AGATTGGG

### Experimental Design and Statistical Analysis

Sample sizes were estimated from pilot studies and previous data using power analysis or the resource equation method to achieve 80% power at *P* < 0.05. Animals were randomized to experimental groups (e.g., time-point after MCAO, drug treatment) using a computer-based random number generator (https://www.randomizer.org/). The assessor was unware of allocation to experimental group during analysis of outcome measures although no formal blinding procedures were in place. Data are presented as mean ± SEM unless otherwise indicated. For normally distributed data, differences were tested using unpaired Student’s *t*-test or analysis of variance (ANOVA) with Bonferroni or Tukey correction unless otherwise stated. For non-normally distributed data, equivalent non-parametric tests were used and results displayed as median ± minimum and maximum values. Data were analyzed using GraphPad Prism. In all experiments, values of *P* ≤ 0.05 were accepted as statistically significant.

## Results

### Increased Density of Macrophages in the RP After Experimental Stroke

**Figure 1:**
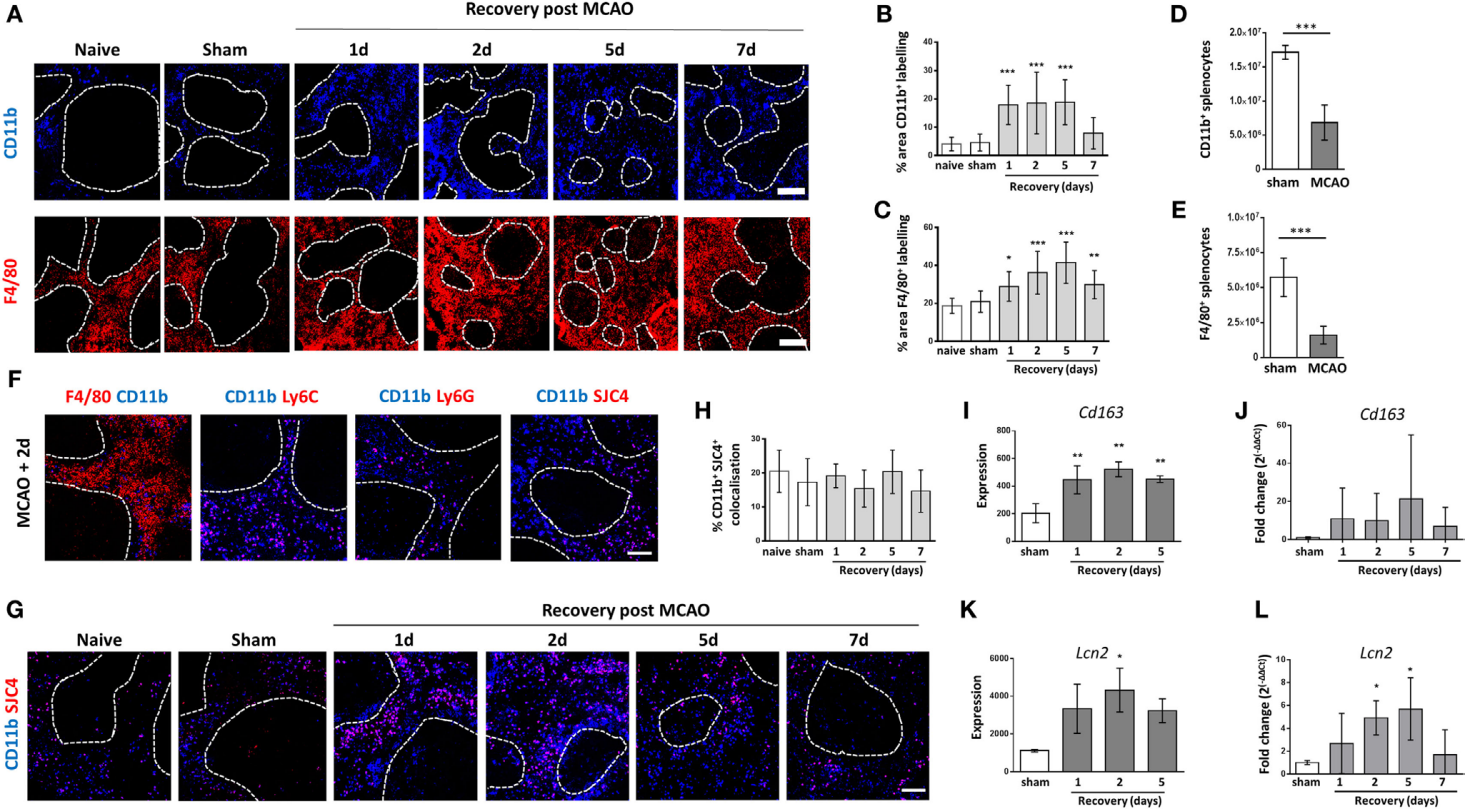
Increased density of macrophages in the red pulp (RP) after experimental stroke. **(A)** Immunofluorescent labeling of monocyte-derived RPM (blue, CD11b) or both tissue resident and monocyte-derived RPM (red, F4/80) in the splenic RP from naïve mice, mice recovered 1 to 7 days after middle cerebral artery occlusion (MCAO) or sham-operated mice. Quantification of immunolabelling shows increased percentage area of CD11b **(B)** and F4/80 **(C)** immunolabelling per section in mice 1–5 days or 1–7 days, respectively, after experimental stroke in comparison to sham-operated or naïve controls (*naïve n* = *4, sham n* = *4, 1 day n* = *4, 2 days n* = *9, 5 days n* = *5, 7 days n* = *7*). Flow cytometry analysis of the spleen shows that the absolute number of **(D)** CD11b^+^ and **(E)** F4/80^+^ cells is reduced 2 days after stroke in comparison to sham-operated animals (*sham n* = *4, MCAO n* = *6*). **(F)** Immunofluorescent labeling of CD11b (blue) alongside markers for macrophages (red, F4/80), monocytes (red, Ly6C), and neutrophils (red Ly6G; SJC4) in spleens from mice recovered 2 days after MCAO (*n* = *4*). **(G)** Immunofluorescent labeling of CD11b (blue) and SJC4 (red) in the splenic RP from naïve mice, mice recovered 1 to 7 days after MCAO or sham-operated mice. **(H)** Quantification of CD11b and SJC4 shows the percentage of CD11b^+^ immunolabelling that co-localizes with SJC4 is not significantly altered 1–7 days after MCAO (*naïve n* = *4, sham n* = *4, 1 day n* = *4, 2 days n* = *9, 5 days n* = *5, 7 days n* = *7*). Analysis of spleen microarray data shows significantly increased transcription of *Cd163* **(I)** at 1–5 days and *Lipocalin2* **(K)** at 2 days after MCAO in comparison to sham-operated controls (*sham n* = *2, 1 day and 2 days n* = *3, 5 days n* = *4*). **(J)** RT-qPCR analysis confirmed a trend for upregulation of *Cd163* mRNA after experimental stroke. **(L)** RT-qPCR analysis of *Lcn2* mRNA confirmed upregulation at 2–5 days after MCAO in comparison to sham operated animals (*naïve n* = *4, sham n* = *4, 1 day n* = *4, 2 days n* = *9, 5 days n* = *5, 7 days n* = *7*). Scale bars **(A)** 200 µm, **(F,G)** 100 µm. Data show mean ± SD **(B,C,H–L)** *P < 0.05; **P < 0.01; ***P < 0.05 one-way analysis of variance with Bonferonni correction; **(D,E)** unpaired *t*-test.

RPM remove senescent erythrocytes from the blood, regulate iron metabolism, and contribute to the cytokine microenvironment, which directs adaptive immune responses (19, 23). Given the importance of these functions for maintaining red blood cell homeostasis, iron recycling and reducing bacterial infection, we investigated specific effects of experimental stroke on RPM populations. Immunofluorescent labeling with F4/80 detects both tissue resident (F4/80^hi^ CD11b^−/lo^) and monocyte-derived (F4/80^+^ CD11b^+^) populations of RPM (described above), whereas labeling of CD11b detects monocyte-derived (F4/80^+^ CD11b^+^) RPM only. Both methods of detecting RPM showed an increased density of macrophages in the RP from 1 to 7 days after experimental stroke (CD11b; blue, F4/80; red, Figure 1A), which is confirmed by analysis of the percentage of positive immunofluorescence for each marker per image (Figures 1B,C). By 5 days after experimental stroke, large clusters of monocyte-derived CD11b^+^ macrophages (CD11b; blue, Figure 1A) were found within the WP, whereas in naïve and sham-operated controls, this subset is confined to the RP only suggesting a loss of the normal boundaries between RP and WP macrophage subsets. Despite this local increase in cell density, flow cytometric analysis showed an overall reduction in the absolute number of RPM in spleens 2 days after experimental stroke in comparison to sham-operated animals (Figures 1D,E). The reduction in cell numbers is most likely due to the splenic atrophy and loss of total cellularity known to occur after stroke resulting in a local increase in RPM density within remaining contracted spleen tissue but no overall increase in cell number.

**Figure 2:**
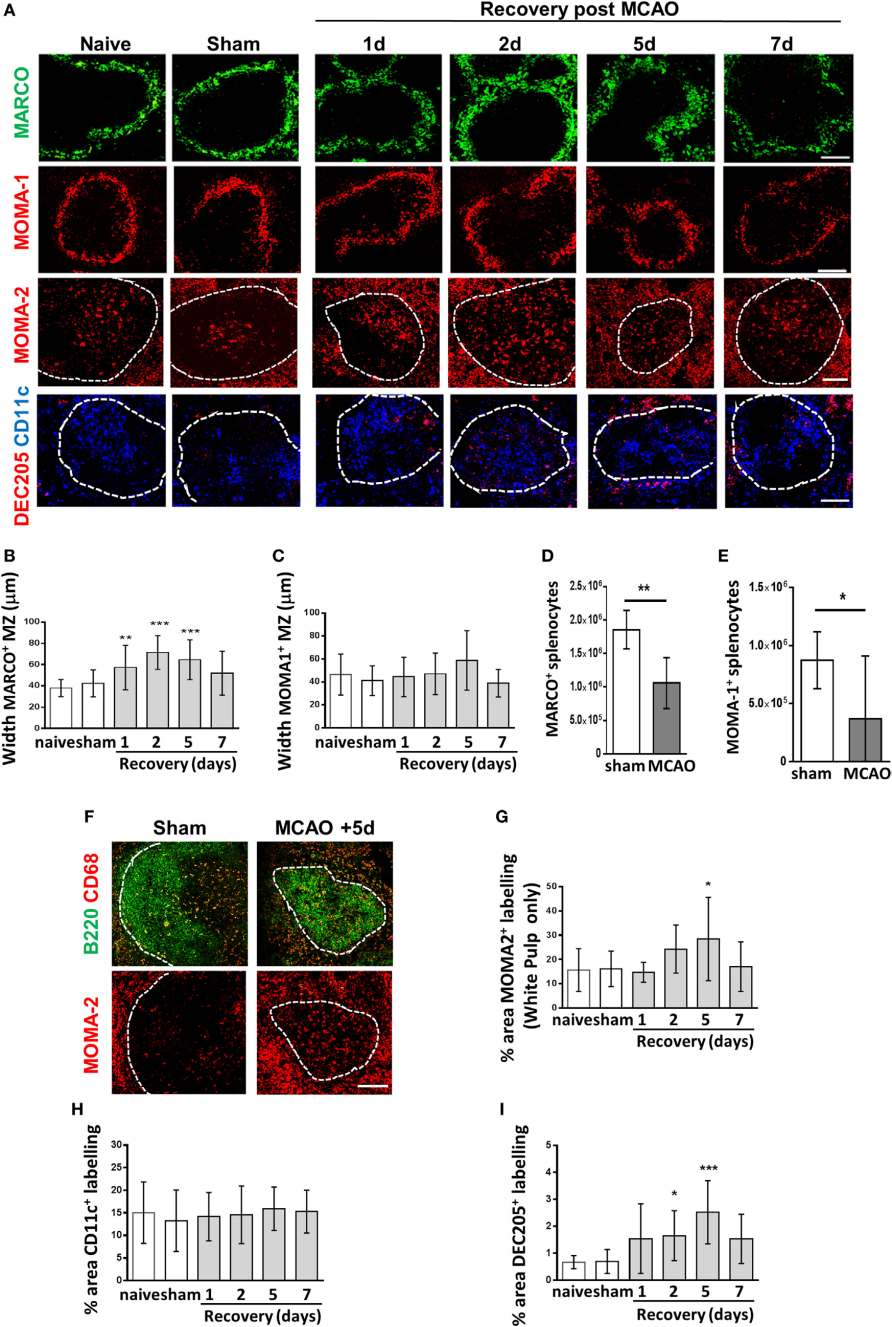
Experimental stroke differentially affects marginal zone (MZ) and white pulp (WP) macrophage populations. **(A)** Immunofluorescent labeling of spleens from mice recovered 1–7 days after middle cerebral artery occlusion (MCAO) for marginal zone macrophages (MZM) (green, MARCO), marginal zone metallophillic macrophages (red, MOMA-1), tingible body macrophages (red, MOMA-2 within WP), classical dendritic cells (blue, CD11c), and DEC205^+^ DC (red, DEC205). **(B)** Measuring the width of MARCO^+^ immunolabelling in the MZ confirms expansion of the MZM population 1–5 days after experimental stroke whereas the width of the MOMA-1^+^ MZ metallophillic macrophage border **(C)** remains unchanged. Flow cytometry analysis of the spleen shows that the absolute number of **(D)** MARCO^+^ and **(E)** MOMA-1^+^ cells are reduced 2 days after stroke in comparison to sham-operated animals (*sham n* = *4, MCAO n* = *6*). **(F)** Immunofluorescent labeling of spleens with CD68 (red) alongside B cells (B220, green) confirms a similar pattern of immunostaining in the WP as MOMA-2 (red) immunolabelling (*naïve n* = *4, sham n* = *4, 1 day n* = *4, 2 days n* = *9, 5 days n* = *5, 7 days n* = *7*). **(G)** Quantification of the percentage of MOMA-2^+^ tingible body macrophage immunolabelling in the WP confirms significant increases in MOMA-2^+^ immunolabelling 5 days after experimental stroke in comparison to sham-operated controls. **(H)** The percentage area of CD11c^+^ immunolabelling is unchanged after experimental stroke in comparison to sham-operated animals. **(I)** A significant increase in the percentage of DEC205^+^ immunolabelling per section can be seen 2–5 days after experimental stroke in comparison to sham-operated controls (*naïve n* = *4, sham n* = *4, 1 day n* = *4, 2 days n* = *9, 5 days n* = *5, 7 days n* = *7*). Scale bars 100 µm. Data show mean ± SD *P < 0.05; **P < 0.01; ***P < *0.005*; **(B,C,G–I)** one-way analysis of variance with Bonferonni correction. **(D,E)** unpaired *t*-test.

**Figure 3:**
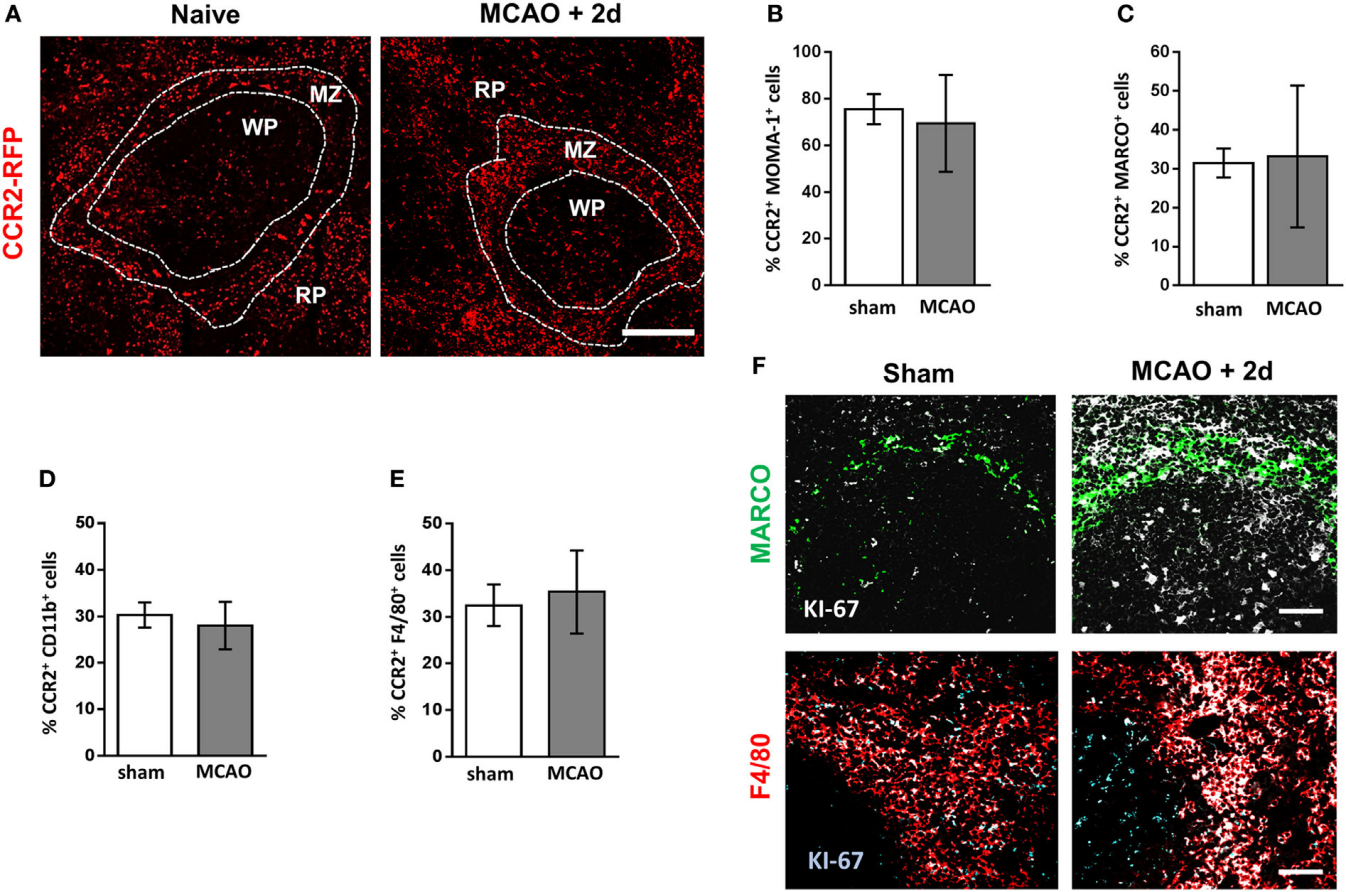
Increased density of splenic macrophage subsets is not due to increased recruitment from bone marrow. **(A)** Spleen sections from naïve CCR2^RFP/+^ mice or CCR2^RFP/+^ recovered 2 days after MCAO to determine number and location CCR2^+^ (red; RFP) cells infiltrating from bone marrow (*naïve n* = *4, MCAO* + *2 days n* = *4*). Flow cytometric analysis of spleens from CCR2^RFP/+^ mice shows no difference in the percentage of **(B)** MOMA-1^+^; **(C)** MARCO^+^; **(D)**; CD11b^+^; or **(E)** F4/80^+^ cells expressing RFP in mice recovered 2 days after MCAO in comparison to sham-operated animals (*sham n* = *4, MCAO n* = *6*). **(F)** Increased immunofluorescent labeling of proliferation markers (white, KI-67) associated with MARCO^+^ marginal zone macrophages (green, MARCO) or red pulp macrophages (red, F4/80) in the spleens of mice recovered 2 days after MCAO in comparison to sham-operated animals (*sham n* = *4, MCAO n* = *4*). Scale bars **(A)** 100 µm, **(F)** 50 µm. Data show mean ± SD; unpaired *t*-test.

CD11b can be expressed by multiple myeloid cell types, therefore to determine the distribution of CD11b immunolabelling on splenocytes, we assessed co-localization of CD11b with F4/80, Ly6C (expressed on monocytes and granulocytes) Ly6G (expressed specifically by neutrophils) and with the antibody SJC4 (neutrophil-specific polyclonal antibody). F4/80^+^CD11b^+^ cells representing the monocyte-derived RPM were detected but F4/80^–^CD11b^+^ cells were also present in the RP indicating that not all of the CD11b^+^ cells were RPM (Figure 1F). Some CD11b^+^ cells co-localized with Ly6C, LygG, and SJC4 suggesting that the F4/80^−^CD11b^+^ cells in the RP were comprised of monocytes and neutrophils (Figure 1F). To determine if the increased density of CD11b^+^ cells demonstrated previously (Figures 1A,B) was due to an increase in immunolabelling of neutrophils, we examined CD11b and SJC4 co-localization after experimental stroke (Figure 1G). The percentage of CD11b^+^SJC4^+^ co-localization was not significantly altered after experimental stroke in comparison to naïve or sham-operated animals (Figures 1G,H). As the area of CD11b^+^ immunolabelling is increased after stroke (Figure 1B) and percentage of CD11b^+^SJC4^+^ co-localization remains unchanged, it can be concluded that the density of F4/80^+^CD11b^+^ monocyte-derived RPM does indeed increase after experimental stroke, and there is a proportional increase in CD11b^+^SJC4^+^ neutrophils also. CD11b is also expressed by subpopulations of DC in the spleen including TNF/iNOS producing (Tip) DC, monocyte-derived (mo) DC, L-DC, and expressed at low levels by conventional CD8^−^ DC (43, 44). Immunolabelling for CD11c, which is expressed by the majority of DC subsets including CD8^−^ DC (43), was quantified and is unaffected by experimental stroke (Figures 2A,H). Additionally, the proportion of CCR2-RFP^+^ CD11b^+^ cells remains unchanged by experimental stroke (Figure 3D) meaning it is unlikely that TipDC and moDC, which are both derived from recruited monocytes under conditions of inflammation contribute to increased CD11b^+^ cells in the spleen after stroke. L-DC make up less than 3% of the total myeloid/DC pool in the spleen (44). Therefore although we have not specifically assessed the contribution of CD11b^+^ DC subsets in this study, these subsets are unlikely to be major contributors to the increased CD11b density seen after experimental stroke.

As number, density, and location of RPM were affected by experimental stroke, we next determined expression levels of genes of interest important for RPM-mediated antibacterial defense from microarray data obtained from spleens from sham-operated animals or animals recovered 1, 2, or 5 days after experimental stroke. CD163, a scavenger receptor that is highly expressed on RPM relative to other macrophage subsets, allows the clearance of hemoglobin released from damaged erythrocytes (23, 45). RPM additionally express Lipocalin 2, which binds the bacterial siderophores pathogens use to scavenge iron. Sequestration of siderophores is important mechanism in preventing bacterial growth and reducing infection (46). Lipocalin2 can also be expressed by neutrophils, which may additionally contribute to splenic antibacterial defenses. Microarray analysis showed increased expression of transcripts for *Cd163* (Figure 1I) and *Lcn2* (Lipocalin2; Figure 1K) 1–5 days after experimental stroke in comparison to sham-operated controls. RT-qPCR analysis of mRNA showed a trend for increased CD163 expression after experimental stroke although this was not statistically significant (Figure 1J). RT-qPCR analysis of *Lcn2* confirmed significant upregulation at 2–5 days after experimental stroke in comparison to sham (Figure 1L). RPM-associated genes are upregulated despite the significant overall reduction in numbers of RPM. Increased local density of neutrophils after experimental stroke may further contribute to this retained antibacterial defense mechanism.

### Experimental Stroke Differentially Affects MZ and WP Macrophage Populations

The highly organized structure of the spleen and the positioning of macrophage sub-populations in distinct micro-anatomical locations are crucial to enable their distinct but complementary roles in antibacterial defense, pathogen/apoptotic cell clearance, and regulation of adaptive immune responses (18, 29, 31, 34, 35, 38, 39). Experimental stroke induced an increase in MZM immunolabelling (MARCO; green, Figure 2A) indicating an increased cellular density. This resulted in a significant thickening of the width of the MZM border measured at 1–5 days after experimental stroke (Figure 2C). In contrast, MMM remained unaffected (MOMA-1; red, Figures 2A,D). Similarly to RPM, absolute numbers of MARCO^+^ and MOMA-1^+^ macrophage subsets were reduced 2 days after experimental stroke in comparison to sham-operated animals (Figures 2E,F). TBM can be identified by their distinct morphology, large cells with tingible bodies within the cytoplasm, and their location within the follicle. MOMA-2^+^ immunolabelling was measured within the WP only to specifically detect TBM and showed increased TBM immunolabelling at 5 days after experimental stroke (MOMA-2; red, Figures 2A,G). Immunolabelling of CD68 and B220 on serial sections was used to confirm TBM immunolabelling which are CD68^+^ and located within the B cell follicle (Figure 2B). CD68 immunolabelling occurred in a similar pattern to MOMA-2 immunolabelling within the WP providing further support that these were indeed TBM (Figure 2F) (47, 48). No difference in the amount of CD11c^+^ immunolabelling was identified (Figure 2H). However, CD11c^+^ DC which are normally enriched in the periarteriolar lymphoid sheath (PALS) area of the WP and the MZ, became dispersed throughout WP at 2 and 5 days after experimental stroke with no particular microanatomical localization (CD11c, blue, Figure 2A). This is in accordance with previous reports on loss of distinction between B and T cell areas within the splenic WP after experimental stroke (8). An increase in immunolabelling of DEC205^+^ DC was seen in both the MZ and WP at 1–7 days after experimental stroke and was confirmed by quantification of immunolabelling (Figure 2I). Overall, these data and analysis of RPM populations (Figure 1) demonstrate that distinct subpopulations of splenic macrophages are differentially affected by experimental stroke in terms of their distribution and localization within the spleen.

### Increased Cellular Density of Splenic Macrophage Subsets Is Not Due to Increased Recruitment From Bone Marrow

After experimental stroke, spleen size is reduced due to an overall loss of cellularity and contraction of the organ (8, 10, 14, 16). There is an extensive depletion of lymphocytes and remaining cells are no longer organized into their distinct microanatomical compartments, crucial for effective immune function (8, 10). In contrast, despite overall reductions in numbers, we have shown macrophage populations are maintained within their immunological niches and some subsets show local increases in density. Under homeostatic conditions, splenic macrophages are maintained by both local proliferation, and to a lesser extent, replenished from bone marrow-derived monocytes (22). However, in conditions of inflammation or cellular depletion, both mechanisms can be used to replenish or indeed augment tissue-resident populations (22). Understanding the origin of locally increased macrophage sub-populations is important so that therapeutic strategies affecting macrophages can be optimally designed to reduce susceptibility to stroke-associated infection. *Ccr2*^RFP/+^ mice have the sequence for red fluorescent protein (RFP) knocked into the locus encoding CCR2 at a heterozygous level resulting in expression of RFP in CCR2 expressing cells but retaining normal CCR2 function (41). Upregulation of CCR2 is important for immune cell efflux from the bone marrow and recruitment to sites of inflammation; therefore, RFP^+^ cells in the spleen represent those recently recruited from the bone marrow (49, 50). Analysis of RFP expression in sham-operated animals indicated a contribution of bone marrow-derived cells within the RP, WP, and MZ that remained relatively unchanged 2 days after experimental stroke (Figure 3A). Within the spleen, lymphocytes, NK cells, and neutrophils can express CCR2 under homeostatic conditions, in addition to expression on monocytes/macrophages and DC (51). Flow cytometry was used to determine the percentage of CCR2^+^ cells within macrophage subpopulations to determine the contribution of infiltrating bone marrow-derived monocytes to this expansion. Flow cytometry confirmed there was no increase in the percentage of RFP-expressing MOMA-1^+^ MMM (Figure 3B), MARCO^+^ MZM (Figure 3C), CD11b^+^ (monocyte-derived) RPM (Figure 3D) or F4/80^+^ (monocyte-derived and tissue-resident) RPM (Figure 3E) populations suggesting that the homeostatic rate of replacement of these cells from bone marrow-derived monocytes is not altered by experimental stroke. We demonstrated above increased local densities of tissue-resident and monocyte-derived RPM and MZM after experimental stroke (see Figures 1 and 2); however, there is no increased presence of RFP^+^ cells above baseline turnover within these populations (Figures 3A,C). Identification of actively proliferating cells using KI-67 immunolabelling showed increased KI-67 staining associated with MARCO^+^ macrophages and F4/80^+^ RPM (Figure 3F). Additionally, large KI-67^+^ cells were observed in the WP consistent with the location and morphology of TBM (Figure 3F). Taken together, these data suggest that, after experimental stroke, there is no change in the numbers of bone marrow-derived monocytes recruited to the spleen above normal homeostatic turnover. Increased labeling of KI-67 in F4/80^+^ RPM, MARCO^+^ MZM, and TBM in the WP suggests that these increases may be associated with local proliferation.

### Macrophage Subpopulation-Specific Increases in Markers of Lysosome Activity After Experimental Stroke

**Figure 4:**
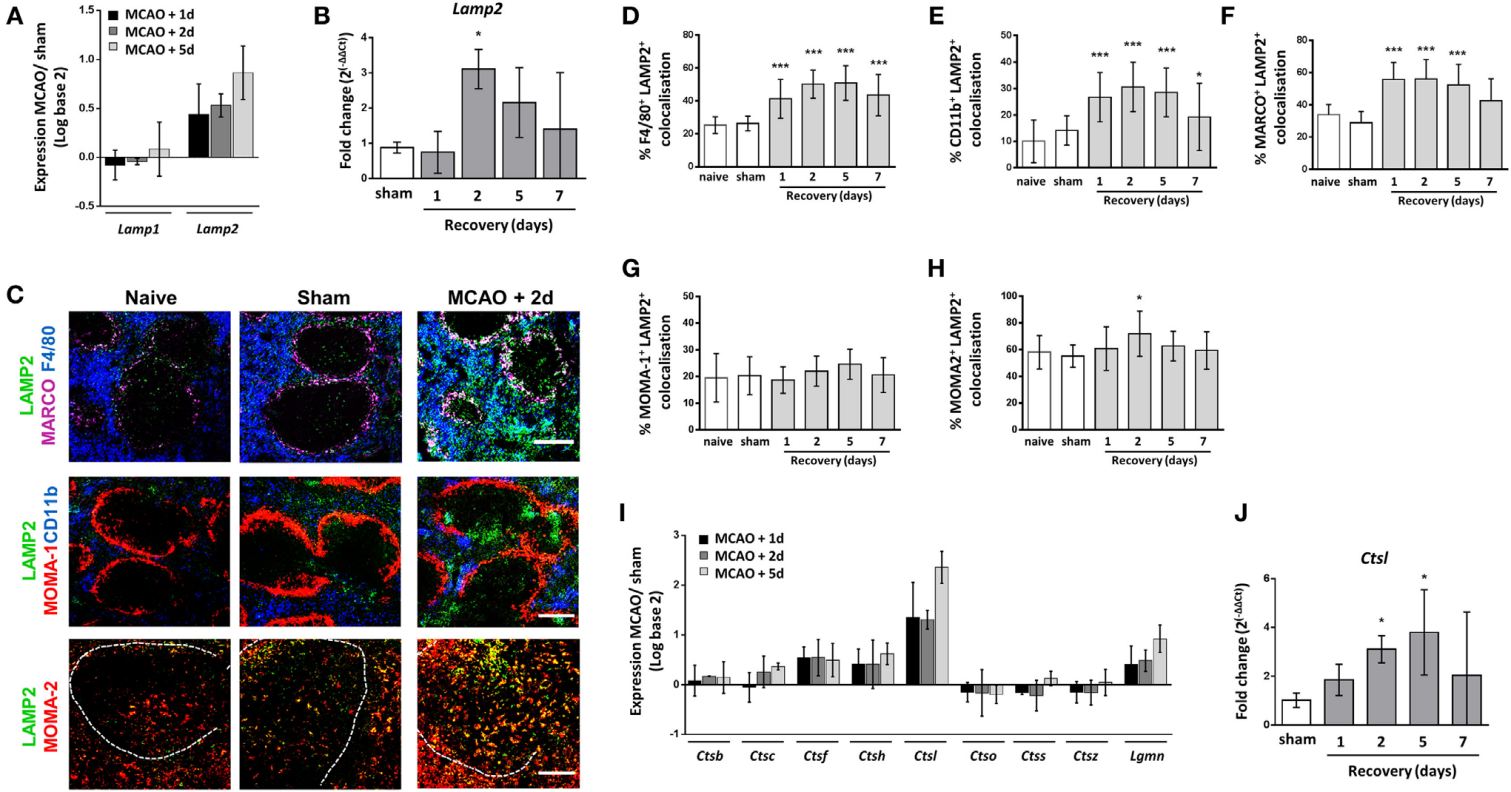
Macrophage subpopulation-specific increases in markers of lysosome activity after experimental stroke. **(A)** Analysis of transcripts of genes associated with lysosome formation show that lysosomal-associated membrane protein 2 (*Lamp2*) expression is upregulated after stroke, whereas *Lamp1* remains relatively unchanged *(sham n* = *2, 1 day and 2 days n* = *3, 5 days n* = *4)*. **(B)** RT-qPCR for *Lamp2* confirmed significant upregulation of expression 2 days after middle cerebral artery occlusion (MCAO) in comparison to sham-operated animals (*naïve n* = *4, sham n* = *4, 1 day n* = *4, 2 days n* = *9, 5 days n* = *5, 7 days n* = *7*). **(C)** Immunofluorescent labeling of LAMP2 (green, LAMP2) along with labeling for both tissue resident and monocyte-derived RPM (blue, F4/80; top panel) marginal zone macrophages (magenta, MARCO; top panel), marginal zone metallophillic macrophages (red, MOMA-1; middle panel), monocyte-derived RPM only (blue, CD11b; middle panel), or tingible body macrophages (red, MOMA-2 within the WP; bottom panel) in spleens from naïve mice, or mice recovered 1–7 days after MCAO or sham surgery (*naïve n* = *4, sham n* = *4, 1 day n* = *4, 2 days n* = *9, 5 days n* = *5, 7 days n* = *7*). Quantification of co-localization of LAMP-2 immunolabelling with macrophage subset markers showed increased expression of LAMP2 associated with **(D)** F4/80^+^ tissue resident and monocyte-derived RPM, **(E)** CD11b^+^ monocyte-derived RPM alone and **(F)** MARCO^+^ MZM at 2–5 days after MCAO in comparison to sham-operated animals. **(G)** However co-localization remained unchanged on MOMA-1^+^ marginal zone metallophillic macrophages **(H)** Co-localization of MOMA-2^+^ immunolabelling with LAMP-2 was high at baseline in naïve and sham-operated animals and was only significantly upregulated at 5 days after MCAO (*naïve n* = *4, sham n* = *4, 1 day n* = *4, 2 days n* = *9, 5 days n* = *5, 7 days n* = *7*). **(I)** Expression of protease transcripts show most proteases are modestly upregulated after MCAO; however, expression of *Ctsl*, which encodes Cathepsin L1, is highly upregulated 1–5 days after experimental stroke in comparison to sham-operated animals *(sham n* = *2, 1 day and 2 days n* = *3, 5 days n* = *4)*. **(J)** RT-qPCR for *Ctsl* confirmed upregulation of expression 2–5 days after MCAO in comparison to sham-operated animals (*naïve n* = *4, sham n* = *4, 1 day n* = *4, 2 days n* = *9, 5 days n* = *5, 7 days n* = *7*). Scale bars **(C)** top panel, middle panel 200 µm; **(C)** bottom panel 100 µm. Data show mean ± SD **(B,D–H,J)** *P < 0.05; ***P < 0.005; one-way analysis of variance with Bonferonni correction.

Phagocytosis of pathogens and senescent cells is a crucial role of many splenic macrophage subsets. Analysis of microarray data from spleen tissue collected 1, 2, or 5 days after experimental stroke was used to assess expression of families of functionally related genes associated with these processes after experimental stroke. LAMP 1 and 2 are expressed on the membrane of lysosomes and are thought to be important in maintaining lysosomal integrity, pH, and catabolism (52). Splenic expression of *Lamp2* but not *Lamp1* was increased after experimental stroke (Figure 4A) and analysis of mRNA levels by RT-qPCR confirmed significant upregulation of *Lamp2* 2 days after experimental stroke in comparison to sham surgery (Figure 4B). To determine the splenic macrophage subsets affected, we analyzed co-localization of splenic LAMP2 immunolabelling alongside macrophage subset-specific markers. Co-localization of LAMP2 immunolabelling with most macrophage subsets was observed to some extent in naïve and sham-operated animals. However, image analysis showed LAMP2 co-localization was significantly increased in both F4/80^hi^ CD11b^−/lo^ and F4/80^+^ CD11b^+^ subsets of RPM (Figures 4C–E) and MARCO^+^ MZM (Figures 4C,F) 2 days after experimental stroke. In accordance with the function of TBM in the clearance of apoptotic cells from the follicle, high levels of LAMP2^+^ immunolabelling co-localized with MOMA-2^+^ cells in the WP of naïve and sham-operated animals and a small increase was detected 2 days after experimental stroke (Figures 4C,H). In contrast, LAMP2 expression by MOMA-1^+^ MMM remained relatively unchanged (Figures 4C,G).

After phagocytosis, proteins are processed into peptides by endo/lysosomal cysteine and aspartate proteases. The protease cathepsin L is specifically involved in generation of peptides for antigen presentation within the mouse spleen (53). An upregulation of transcript for cathepsins, including Cathepsin L (*Ctsl*) and Legumain (*Lgmn*) was seen in spleens 1–5 days after experimental stroke in comparison to sham-operated controls (Figure 4I) and increased expression of *Ctsl* mRNA was confirmed by RT-qPCR suggesting there was no impairment in proteolytic processing of endocytosed material in the spleen after experimental stroke (Figure 4J).

These data show upregulation of genes encoding proteins involved in lysosome function and proteolytic processing after experimental stroke and a subset-specific increase in a key marker of macrophage lysosome activity was confirmed by immunohistochemical labeling of LAMP2 in both tissue-resident and bone marrow-derived RPM and MZM. This suggests that there are no impairments in these processes, which are crucial for efficient phagocytosis, in the spleen due to experimental stroke.

### MZM Efficiently Traffic Antigen From the Circulation to the Follicle

**Figure 5:**
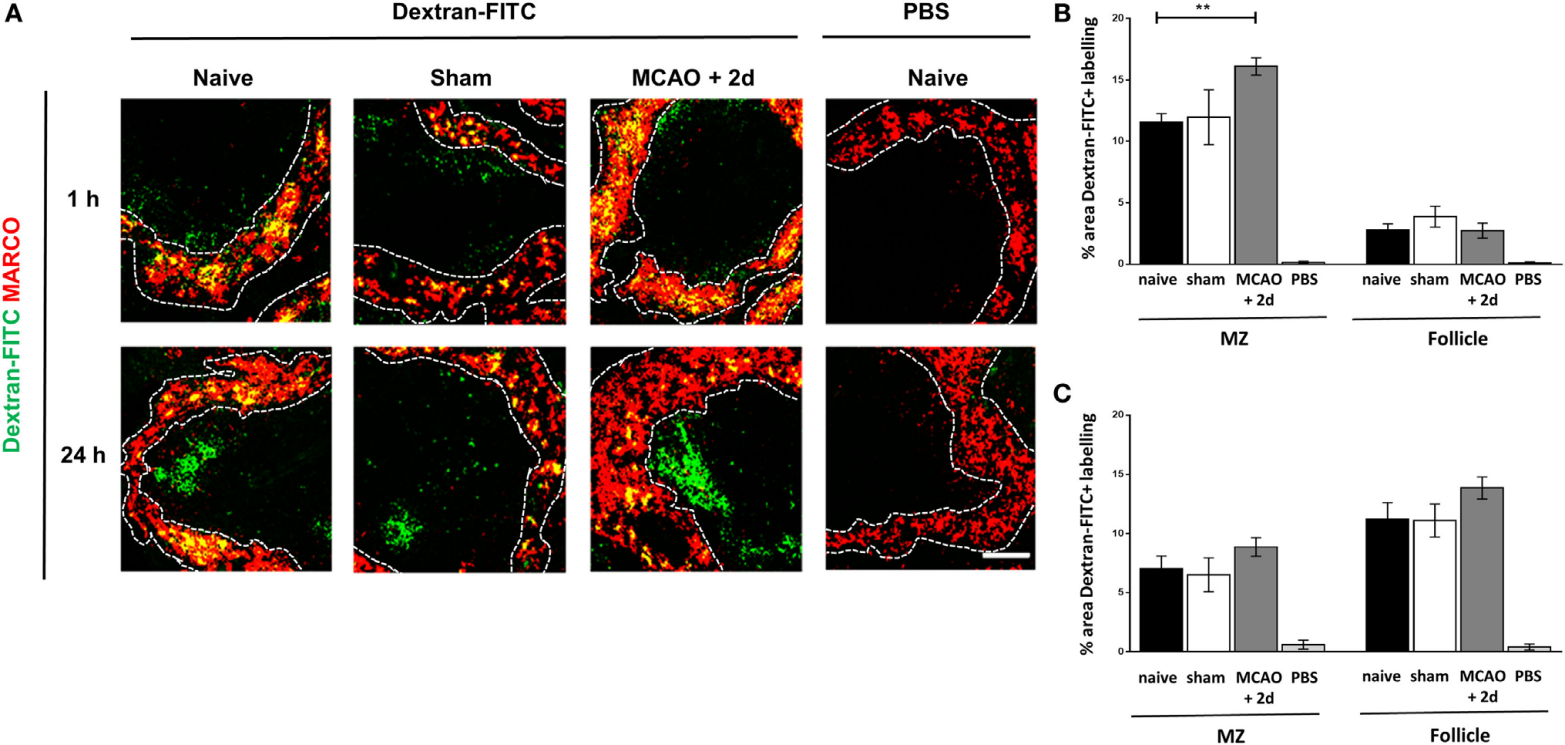
Marginal zone macrophages (MZM) efficiently traffic antigen from the circulation to the follicle. **(A)** Fluorescently labeled model antigen (green, Dextran-FITC) is trapped in the spleen by MZM (red, MARCO) in spleens from animals recovered 2 days from middle cerebral artery occlusion (MCAO) or sham surgery or naïve controls 1 h after iv injection. By 24-h post iv injection, antigen has been trafficked into the follicle of the white pulp (WP). Naïve animals were injected with vehicle (PBS) as a control. Quantification of the percentage area of Dextran-FITC associated with the marginal zone (MZ) or WP of the spleen at 1 h **(B)** and 24 h **(C)** shows increased trapping of antigen in the MZ at 1 h and no impairments in antigen trafficking to the follicle at 24 h in mice 2 days after MCAO in comparison to sham or naïve controls (for each time point *naïve n* = *4, sham n* = *2, MCAO n* = *4, naïve* + *PBS n* = *2*). Scale bar 50 µm. Data show mean ± SD **(B,C)**, **P < 0.01; one-way analysis of variance with Bonferonni correction.

The transport of intact protein antigen from the MZ to the FDC network is crucial to aid the generation of high affinity antibody production by B cells (54, 55). Given the increased expression of genes associated with lysosome activity and proteolytic processing and the increased LAMP2 staining in MZM (see Figure 4), we next determined if there were any impairments in antigen trafficking from the MZ to the WP after experimental stroke. Spleens were harvested 1 h after injection of fluorescently labeled dextran to determine initial trapping in the MZ and at 24 h after injection to determine subsequent trafficking to the follicle. Increased capture of Dextran-FITC associated with MARCO^+^ MZM was observed 2 days after experimental stroke in comparison to sham-operated and naïve controls (Figures 5A,B). This could relate to the increased density of MARCO^+^ MZM present after experimental stroke (Figures 2A,B). At this time point, only low levels of Dextran-FITC were detected in the WP, and this was similar across all groups (Figures 5A,B). At 24 h after injection, antigen was efficiently transported into the follicle and levels were similar across all groups (Figures 5A,C). These data indicate the trafficking of intact antigen is not impaired by the increased endosomal and proteolytic activity detected in MARCO^+^ MZM after experimental stroke. This implies the ability of macrophages specialized to capture and traffic antigen to the FDC network and facilitate the GC response is not impaired by experimental stroke.

### Macrophage Subpopulation-Specific Impairments in Expression of MHC Class II

**Figure 6:**
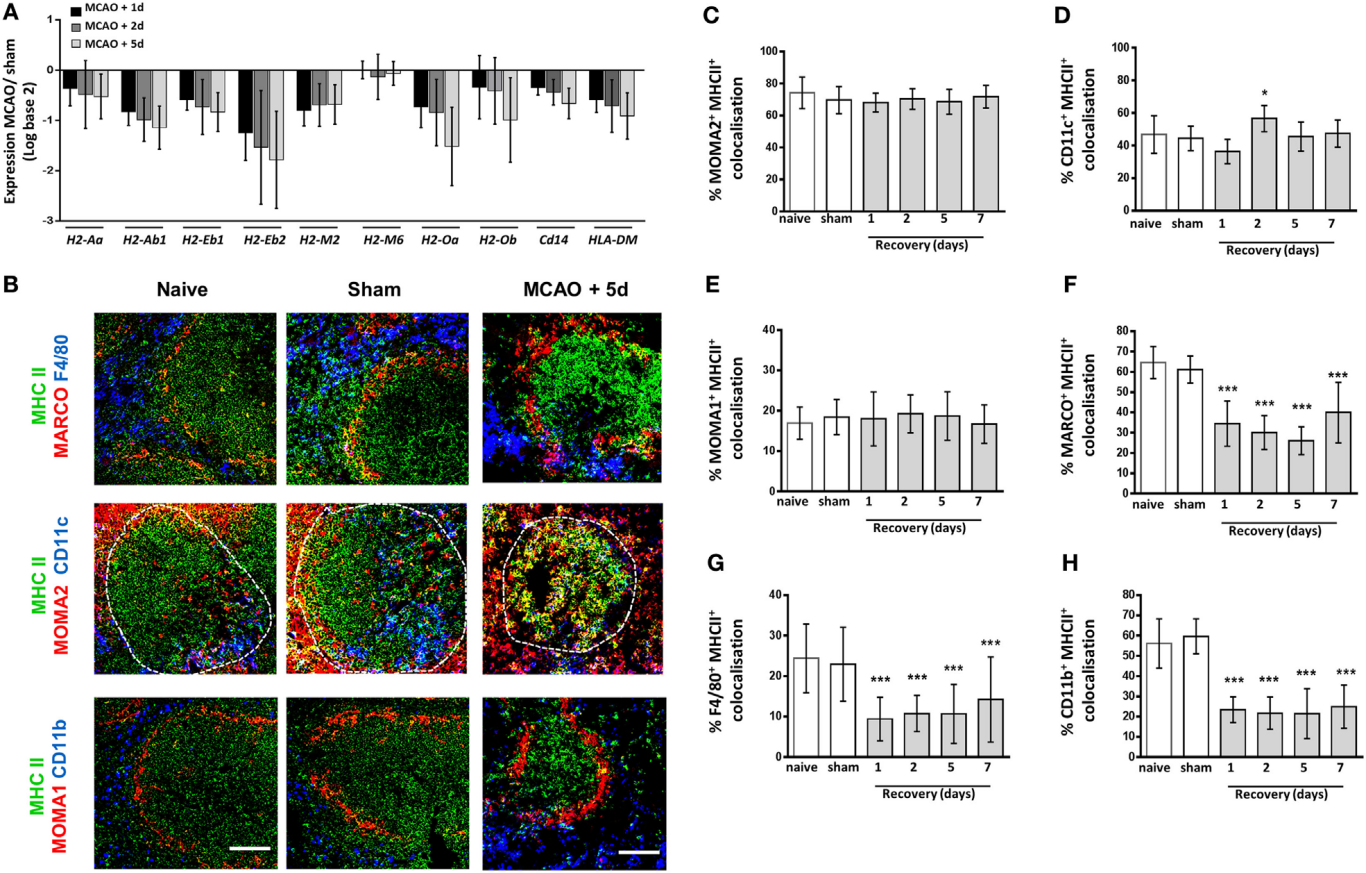
Macrophage subpopulation-specific impairments in expression of MHC Class II. Genome-wide transcriptional analysis was performed by microarray of RNA extracted from spleen tissue of mice recovered 1, 2, or 5 days after middle cerebral artery occlusion (MCAO) or sham surgery. The fold-change of macrophage-associated gene expression levels was analyzed in relation to expression measured in spleens from sham-operated animals **(A)** Analysis of the *H2* genes which encode mouse MHC class II show downregulation of expression of all *H2* genes 1–5 days after MCAO in comparison to sham-operated animals *(sham n* = *2, 1 day and 2 days n* = *3, 5 days n* = *4)*. **(B)** MHC Class II (green) immunolabelling along with labeling for marginal zone macrophages (MZM) (red, MARCO; top panel), both tissue resident and monocyte-derived RPM (blue, F4/80 top panel), tingible body macrophages (red, MOMA-2 within white pulp; middle panel), classical dendritic cells (blue, CD11c middle panel), marginal zone metallophillic macrophages (red, MOMA-1; bottom panel) or monocyte-derived RPM only (blue, CD11b; bottom panel) to determine co-localization of MHC Class II on splenic macrophage subsets. Image analysis determined the percentage of **(C)** MOMA-2, **(D)** CD11c, **(E)** MOMA-1, **(F)** MARCO, **(G)** F4/80, and **(H)** CD11b immunolabelling that co-localized with MHC class II immunolabelling 1–7 days after MCAO in comparison to sham-operated or naïve controls (*naïve n* = *4, sham n* = *4, 1 day n* = *4, 2 days n* = *9, 5 days n* = *5, 7 days n* = *7*). Scale bars 100 µm. Data show mean ± SD *P < 0.05; ***P < 0.005; **(C–H)** one-way analysis of variance with Bonferonni correction.

Previous studies have reported reduced expression of MHC class II on human blood monocytes and mouse splenocytes after stroke, which demonstrates a reduced activation status and an inability to present antigen to aid adaptive immune responses (11, 12, 56, 57). However, it is currently unknown if all macrophage subsets are equally affected. Analysis of microarray data was used to assess expression of genes related to MHC class II expression, stability and peptide loading. This analysis demonstrated reduced expression of the *H2* genes, *H2-Aa, H2-Ab1, H2-Eb1, H2-Eb2, H2-M2, H2-M3, H2-Oa*, and *H2-Ob*, responsible for MHC Class II expression in comparison to spleens from sham-operated animals (Figure 6A). Furthermore, expression of genes encoding proteins involved in stabilization of MHC Class II molecules, the MHC Class II invariant chain (*Cd74*; Figure 6A), and loading of peptide for antigen presentation (*HLA-DM*; Figure 6A) were also downregulated after experimental stroke. To determine if reduced expression of MHC Class II could be associated with specific splenic macrophage subsets, MHC Class II immunolabelling was performed alongside various macrophage subpopulation markers and the percentage of each subset marker that co-localized with MCH class II immunolabelling was quantified. This demonstrated MHC class II expression by MOMA2^+^ TBM (red, Figures 6B,C) within the WP was unchanged after experimental stroke. A small increase in MHC class II co-localization with CD11c^+^ classical DC immunolabelling was detected at 2 days post experimental stroke but remained unchanged at all other time points (blue; Figures 6B,D). Low levels of co-localization of MHC Class II with MOMA1^+^ MZM was detected in spleens from sham and naïve animals and again this was unchanged in response to experimental stroke (red Figures 6B,E). However, expression of MHC Class II on MARCO^+^ MZM (red; top panel, Figures 6B,F) and both populations of RPM (F4/80; blue, CD11b; blue; Figures 6B,G–H) appeared reduced 1–7 days after experimental stroke. MHC Class II immunofluorescence within the WP that is not co-localized with any macrophage marker was also reduced after experimental stroke. This most likely represents MHC Class II^+^ B cells which we have previously shown to be severely depleted after experimental stroke (8). These data suggest that impairments in the expression of MHC Class II specifically occurs in RPM and MZM after experimental stroke, whereas other subsets such as TBM, DC, and MMM remain unaffected.

### Reduced Splenic Expression of Genes Required for Macrophage Response to Pathogens

**Figure 7:**
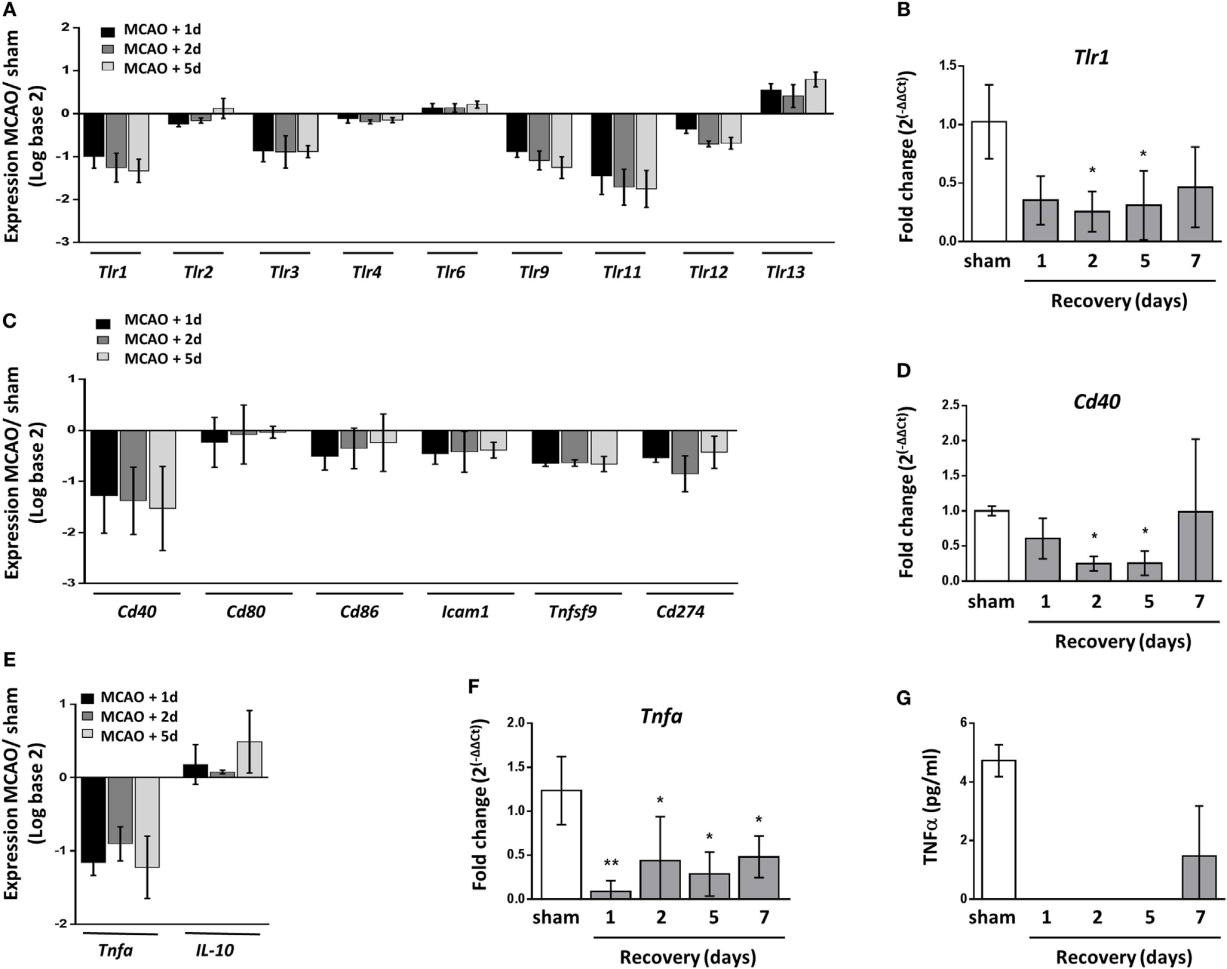
Reduced splenic expression of genes required for macrophage response to pathogens. **(A)** Analysis of toll-like receptor (TLR) transcripts show downregulated expression of the majority of *tlr* genes 1–5 days after middle cerebral artery occlusion (MCAO) in comparison to sham-operated animals *(sham n* = *2, 1 day and 2 days n* = *3, 5 days n* = *4)*. **(B)** RT-qPCR for *Tlr1* confirmed significant downregulation at 2–5 days after MCAO in comparison to sham-operated animals (*naïve n* = *4, sham n* = *4, 1 day n* = *4, 2 days n* = *9, 5 days n* = *5, 7 days n* = *7*). **(C)** Expression of transcripts which encode macrophage-associated co-stimulatory proteins important for activating the adaptive immune response show downregulated expression 1–5 days after MCAO in comparison to sham-operated animals *(sham n* = *2, 1 day and 2 days n* = *3, 5 days n* = *4)*. **(D)** RT-qPCR for *Cd40* confirmed significant downregulation at 2–5 days after MCAO in comparison to sham-operated animals (*naïve n* = *4, sham n* = *4, 1 day n* = *4, 2 days n* = *9, 5 days n* = *5, 7 days n* = *7*). **(E)** Expression of *Tnfa* which encodes the cytokine TNFα was downregulated 1–5 days after experimental stroke whereas expression of *Il-10* which encodes the cytokine IL-10 remained relatively unchanged *(sham n* = *2, 1 day and 2 days n* = *3, 5 days n* = *4)*. **(F)** RT-qPCR for *Tnfa* confirmed downregulation of expression 1–7 days after MCAO in comparison to sham-operated animals (*naïve n* = *4, sham n* = *4, 1 day n* = *4, 2 days n* = *9, 5 days n* = *5, 7 days n* = *7*). **(G)** CBA assay of homogenized spleen detected TNFα in the spleens of sham-operated animals and animals recovered 7 days after MCAO; however, this was undetectable in spleens from mice recovered 1–5 days after MCAO. Data show mean ± SD **(B,D,F)**, *P < 0.05; **P < 0.01; one-way analysis of variance with Bonferonni correction.

Functional deactivation of monocytes/macrophages after stroke is thought to contribute to SAI with deficits reported in activation of adaptive immune responses and production of inflammatory cytokines (11, 12, 56–58). Transcriptional analysis showed a reduction in the expression of toll-like receptor (TLR) genes (Figure 7A), and this finding was validated for *Tlr1* by RT-qPCR (Figure 7B). Many splenic cell populations can express TLR however cells of the innate immune system are reported to express a broader range of TLR than that of the adaptive immune system (59). These data suggest there is a reduced ability to detect pathogens through TLR ligation in the spleen after stroke which may impact macrophage ability to detect and respond to pathogen.

Human blood monocytes and mouse splenocytes have also been reported to be less capable of providing co-stimulatory signals required for activating T cells after stroke (56, 57). However as of yet, expression of costimulatory genes or proteins on macrophages after stroke has not yet been assessed. Transcriptional analysis confirmed a reduction in expression of co-stimulatory genes in the spleen known to be expressed by macrophages 1–5 days after experimental stroke including *Cd40, Cd80, Cd86, Icam1, Tnfsf9*, which encodes 4-1BBL, and *Cd274* (Figure 7C). RT-qPCR analysis of *Cd40* mRNA validated these transcriptional findings (Figure 7D). The functional deactivation of human blood monocytes has been further described by reduced production of the cytokine TNFα after stimulation which is thought to be due to increased circulating IL-10 concentrations (10, 11, 58, 60). Transcript levels of *Tnfa* were also reduced in the spleens of mice 1–5 days after experimental stroke in comparison to sham-operated controls whereas *Il-10* transcript was marginally increased above baseline (Figure 7E). RT-qPCR analysis validated reduced *Tnfa* mRNA 1–7 days after experimental stroke (Figure 7F) and levels of TNFα protein were suppressed 1–5 days after experimental stroke using a cytometric bead array on spleen homogenate (Figure 7G).

Overall, these data suggest that the effects of experimental stroke are restricted to RPM populations and MZM specifically whereas other macrophage populations remain unaffected. These subsets are shown to be locally increased and associated with increased expression of lysosomal protein (LAMP2) indicative of increased phagocytic activity. Despite increased expression of genes associated with lysosome activity and proteolytic processing of protein, these same macrophage subsets have a reduced ability to stably produce and express MHC Class II. Further transcriptional data shows reduced splenic expression of genes associated with pathogen sensing and co-stimulation and reduced production of the pro-inflammatory cytokine TNFα. Taken together, these impairments may contribute to a reduced ability to clear pathogens after stroke and an increased susceptibility to SAI.

## Discussion

The data presented here demonstrate that experimental stroke has differential effects on subpopulations of splenic macrophages that are distinct in function, origin and micro-anatomical location. Key findings demonstrate that after experimental stroke in mice:

(1) At a micro-anatomical level, there is increased density of specific subsets of splenic macrophages including both tissue-resident and bone marrow-derived populations of RPM and MZM, whereas other subsets remain unchanged.
(2) These increases are not due to increased recruitment of cells from the bone marrow but may be associated with increased local proliferation of cells within a contracted spleen size.
(3) Macrophage transport of antigen from MZ to follicle is unaffected by these changes.
(4) RPM and MZM selectively show increased evidence of phagocytosis *via* increased LAMP2 expression but reduced ability to present antigen as determined by reduced expression of MHC Class II.

Thus, there is evidence to support subset-selective changes to splenic macrophages after experimental stroke that may contribute to susceptibility to SAI. Furthermore, we demonstrated reduced expression of genes in the spleen relating to sensing of pathogens, co-stimulation of adaptive immune responses and production of the pro-inflammatory cytokine TNFα, which may additionally contribute to impaired anti-bacterial activity after stroke.

The organization of cells into defined micro-anatomical compartments within the spleen is crucial to allow it to carry out its immunological functions effectively (18, 34). Highly adapted sub-populations of macrophages in distinct compartments within the spleen are of variable origin and function but contribute in complementary ways to ensure maintenance of normal homeostasis and efficient protection from infection (61). We have shown differential effects on these subpopulations after experimental stroke. Although there is an overall reduction in spleen size and cellularity after experimental stroke, both populations of RPM were increased in density within the remaining contracted tissue, and we observed CD11b^+^ macrophages normally restricted to the RP appear within the WP after experimental stroke (Figures 1A–C).

Within the MZ, MZM increased in density and there were more DEC205^+^ DC present, but there was no apparent change to MMM (Figures 2A,C,F). In the WP, TBM immunolabelling was increased, but no change in DC density was detected, although their localization was altered (Figures 2A,D,E). It is important to understand stroke induced changes to macrophage subpopulations in the context of their surrounding immune environment as the structural organization facilitates communication between immune cells and allows efficient responses to be generated. However, the limitations of this approach mean that it can be difficult to further dissect precise mechanisms responsible for inducing these changes on specific subpopulations of macrophages and their functional consequences on infection susceptibility in relation to the contribution of other immune cells. We now know there are multiple stimuli present in the spleen after experimental stroke that may contribute to producing these subpopulation specific effects on splenic macrophages, including but not limited to systemic infection, increased levels of stress-associated mediators such as noradrenaline and cortisol (11, 62), and large numbers of apoptotic cells (8). In agreement to our observations on splenic macrophage phagocytosis, *in vitro* exposure of healthy human monocytes to catecholamines, glucocorticoids, or acetylcholine has been shown to have no adverse effects on phagocytosis (63). However, catecholamines and glucocorticoids reduced the ability of monocytes and granulocytes to undergo oxidative burst, a crucial mechanism to clear bacteria internalized by phagocytosis (63). It has also been reported that catecholamines and glucocorticoids reduce NETosis in human neutrophils (64). Understanding which of the multiple stimuli present in the spleen after stroke triggers the responses we see in individual splenic macrophage subpopulations, and why only some populations respond to these, remains to be investigated and may improve our ability to therapeutically target macrophages to improve stroke outcome.

Spleen macrophages are crucial for the recognition and clearance of pathogens in the circulation (27–29, 34). We observed reduced expression of genes in the spleen associated with macrophage activation status (MHC Class II; Figure 6A) and pathogen recognition ability (TLRs Figure 7A), suggesting there may be impairments in the detection of pathogens by TLRs and subsequent activation induced by their ligation. However, recognition of bacterial glycans by macrophage-expressed lectins appears unaffected as indicated by efficient trapping of the model antigen dextran in the MZ after experimental stroke (Figure 5) and our previous reports that expression of the C-type lectin SIGN-R1 by MZM is unaffected by experimental stroke (8). In contrast to reductions in genes associated with macrophage activation, expression of genes associated with phagocytosis through lysosomes, *Lamp2* (Figure 4A), and proteolytic processing of phagocytosed material, *Ctsl* (Figure 4I) were upregulated after experimental stroke suggesting that mechanisms necessary for subsequent phagocytic clearance of pathogens may be unaffected by stroke.

Increased expression of LAMP2 immunolabelling was specifically associated with RPM and MZM but remained relatively unchanged in MMM (Figures 4C–G) showing a subset-specific increase in lysosome activity after experimental stroke. This suggests increases in levels of phagocytosis; however, it remains to be investigated if this is sufficient to clear pathogens and high levels of apoptotic cells present in the spleen after experimental stroke without further detrimental effects. In models of sickle cell anemia, excessive ingestion of erythrocytes led to deficits in macrophage antibacterial function; therefore, it is possible that excessive clearance of apoptotic lymphocytes after experimental stroke could induce similar impairments in splenic macrophage ability to clear pathogens (65). Furthermore, inefficient clearance of apoptotic cells is known to contribute to systemic autoimmune disease in humans and mice (36, 66–68). TBM in the follicle are important for phagocytosing apoptotic lymphocytes from the GC reaction and consistent with this showed high levels of LAMP2 immunolabelling in naïve and sham-operated animals, which was marginally increased by experimental stroke (Figure 4H). However, macrophages in the MZ are additionally important in the clearance of apoptotic cells and the suppression of autoimmune responses to these (31, 35). In the absence of MZ macrophages, clearance is carried out by TBM and RPM with associated increases in pro-inflammatory cytokine production and incidence of autoimmune disease (31). After experimental stroke, we observed increased expression of the lysosomal marker, LAMP2, associated with RPM and MZM (Figures 4D–H) and the presence of CD11b^+^ macrophages within the WP (Figure 1A). This may imply that MZ macrophages and TBM alone are unable to clear the high numbers of apoptotic cells present in the spleen after experimental stroke resulting in local increases in RPM and MZM with increased phagocytic activity. As disruption of subset-specific splenic macrophage function has been related to aberrant regulation of adaptive immune responses and autoimmune disease (19, 21, 31, 36), it remains to be investigated whether the effects of experimental stroke on splenic macrophages results in similar long-term immune dysregulation.

Macrophages can recruit and activate both innate and adaptive immune cells to induce effective immune responses against pathogens. Immunohistochemical analysis indicated splenic macrophages have reductions in expression of MHC Class II suggesting impairments in antigen presentation (Figure 6A). This is comparable to data from monocytes isolated from stroke patients which showed reduced HLA-DR expression in comparison to those isolated from healthy controls (69). Furthermore splenic gene expression analysis showed reduced expression of genes associated with co-stimulation of adaptive immune responses (Figure 7C) and pro-inflammatory cytokine production (Figure 7E) suggesting macrophage ability to directly present antigen to cells of the adaptive immune system is likely to be impaired. We additionally showed that reduced expression of MHC class II occurred in a subset specific manner and that splenic macrophage subsets associated with increased phagocytosis after experimental stroke were also associated with reduced capacity to present antigen on MHC Class II. This may indicate activation of mechanisms designed to reduce autoreactive responses to self-antigen from phagocytosis of apoptotic cells. Gene expression data indicated that deficits in antigen presentation were unlikely to be associated with deficits in phagocytosis or the ability to generate peptide antigen via processing by cathepsins as genes associated with these processes (*Lamp2, Ctsl*) were upregulated after experimental stroke (Figure 4). Instead this was more likely to be due to reduced expression of genes involved in expression of MHC class II in addition to those involved in stabilizing the MHC Class II invariant chain (*Cd74*) and loading complete MHC Class II molecules with peptide (*HLA-DM;* Figure 6). However, cells with more potent antigen presenting and adaptive immune cell activation abilities, such as CD11c^+^ DC, showed no reduction in MHC Class II immunolabelling suggesting some capacity for antigen presentation and activation of adaptive immune responses within the spleen still remains (Figures 6B,D). Macrophages additionally contribute to adaptive immune responses *via* the capture of circulating antigen in the MZ and subsequent transport of intact antigen to FDC in the B cell follicle; an essential mechanism for the selection of high affinity B cells in the GC reaction (33, 55). Despite increased phagocytic activity of MZM (Figure 4), antigen capture by MZM and transport to the follicle was unaffected by experimental stroke (Figure 5) suggesting that macrophage provision of intact antigen to the FDC network to facilitate B cell affinity maturation is unaffected. Infections frequently occur within the first week after stroke onset; therefore, reported deficits in macrophage activation of adaptive immune responses, which are slowly activated, are unlikely to account for early susceptibility to infection but instead may contribute to an inability to clear infection at later stages (13, 70, 71).

Previous reports have shown peripheral monocytes from stroke patients are less able to produce the inflammatory cytokine TNFα upon *in vitro* stimulation (12). In agreement with this, concentrations of TNFα were reduced in spleens after experimental stroke (Figures 7E–G). In combination with deficits in co-stimulatory molecule and class II MHC gene expression, this suggests macrophage ability to communicate with and activate other immune cells, by both direct contact and soluble mediators such as cytokines, was compromised. Macrophage recruitment and activation of innate immune cells may contribute to patient susceptibility to infection early after stroke in addition to deficits in inducing later adaptive immune responses.

In conclusion, our study reveals that subpopulations of splenic macrophages are differentially affected by experimental stroke reflecting the heterogeneity of macrophages within the spleen. Furthermore, although some evidence of functional deactivation was observed, this did not affect all macrophage subsets and other effector functions remained intact, or even augmented, after experimental stroke. Further functional studies to definitively assess the capabilities of individual splenic macrophage subpopulations after stroke are warranted to fully understand the implications of these changes to infection susceptibility after stroke. The heterogeneity in response will be important to consider when investigating therapeutic approaches to combat SAI. For example, deficits in MHC class II expression on monocytes/macrophages has been widely reported; however, non-specific augmentation of MHC Class II expression on all macrophage subsets could increase expression on subsets that do not show any deficit after experimental stroke and push responses toward an autoimmune phenotype with detrimental results long term. In conclusion, selected systemic macrophage functions may provide potential therapeutic targets to reduce SAI and improve clinical outcome; however, it is likely that a subset-targeted approach will be optimal.

## Ethics Statement

All animal experiments were carried out under the authority of a UK Home Office Project Licence in accordance with the “Animals (Scientific procedures) Act 1986” and Directive 2010/63/EU and were approved by both The Roslin Institute’s and the University of Edinburgh’s Animal Welfare and Ethics Review Board. Experimental design, analysis, and reporting followed the ARRIVE guidelines (https://www.nc3rs.org.uk/arrive-guidelines).

## Author Contributions

LM and BM conceived the study. LM, AA, and BM participated in the acquisition of data. LM and BM performed data analyzes. LM and BM drafted the manuscript with assistance from all authors. All authors gave the final approval for publication.

## Conflict of Interest Statement

The authors declare that the research was conducted in the absence of any commercial or financial relationships that could be construed as a potential conflict of interest.

## Abbreviations

DC, dendritic cells; FDC, follicular dendritic cells; MARCO, macrophage receptor with a collagenous structure; MMM, marginal zone metallophillic macrophage; MZ, marginal zone; MZDC, marginal zone dendritic cell; MZM, marginal zone macrophage; PALS, peri-arteriolar lymphoid sheath; PRR, pattern recognition receptor; RP, red pulp; RPM, red pulp macrophage; SAI, stroke-associated infection; TBM, tingible body macrophage; TLR, toll-like receptor; WP, white pulp.
