# Supplementary figures and images for: Experimental Stroke Differentially Affects Discrete Subpopulations of Splenic Macrophages
Source: Front Immunol. 2018 May 22;9:1108. doi: 10.3389/fimmu.2018.01108 (PMC5972287; doi:10.3389/fimmu.2018.01108)

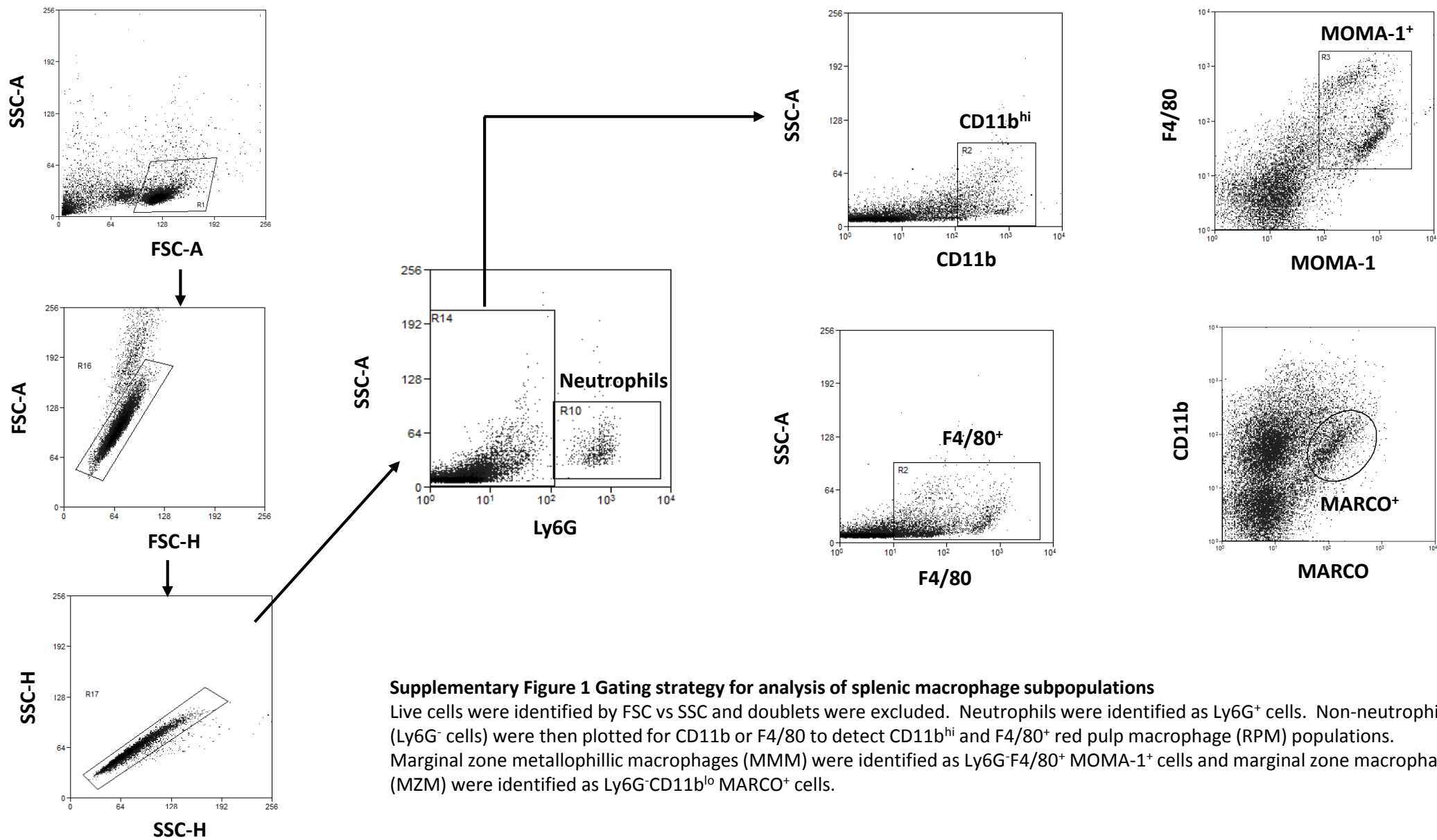

Supplement: Supplementary file 1 [file data_sheet_1.PDF]
